# Metformin produces growth inhibitory effects in combination with nutlin-3a on malignant mesothelioma through a cross-talk between mTOR and p53 pathways

**DOI:** 10.1186/s12885-017-3300-y

**Published:** 2017-05-02

**Authors:** Kengo Shimazu, Yuji Tada, Takao Morinaga, Masato Shingyoji, Ikuo Sekine, Hideaki Shimada, Kenzo Hiroshima, Takao Namiki, Koichiro Tatsumi, Masatoshi Tagawa

**Affiliations:** 1 Department of Respirology, Graduate School of Medicine, Chiba University, 1-8-1 Inohana, Chuo-ku, Chiba, 260-8670 Japan; 2 Division of Pathology and Cell Therapy, Chiba Cancer Center Research Institute, 666-2 Nitona, Chuo-ku, Chiba, 260-8717 Japan; 3 Department of Japanese-Oriental Medicine, Graduate School of Medicine, Chiba University, 1-8-1 Inohana, Chuo-ku, Chiba, 260-8670 Japan; 4 Division of Respirology, Chiba Cancer Center, 666-2 Nitona, Chuo-ku, Chiba, Chiba 260-8717 Japan; 5 Department of Medical Oncology, Faculty of Medicine, University of Tsukuba, Tennodai 1-1-1, Ibaragi, Tsukuba, 305-8575 Japan; 6 Department of Surgery, School of Medicine, Toho University, 6-11-1 Oomori-nishi, Oota-ku, Tokyo, 143-8541 Japan; 7 Department of Pathology, Tokyo Women’s Medical University Yachiyo Medical Center, 477-96 Ohwadashinden, Yachiyo, Chiba, 276-8524 Japan; 8 Department of Molecular Biology and Oncology, Graduate School of Medicine, Chiba University, 1-8-1 Inohana, Chuo-ku, Chiba, 260-8670 Japan

**Keywords:** Mesothelioma, Metformin, Nutlin-3a, p53, Mammalian target of rapamycin

## Abstract

**Background:**

Mesothelioma is resistant to conventional treatments and is often defective in p53 pathways. We then examined anti-tumor effects of metformin, an agent for type 2 diabetes, and combinatory effects of metformin and nutlin-3a, an inhibitor for ubiquitin-mediated p53 degradation, on human mesothelioma.

**Methods:**

We examined the effects with a colorimetric assay and cell cycle analyses, and investigated molecular events in cells treated with metformin and/or nutlin-3a with Western blot analyses. An involvement of p53 was tested with siRNA for p53.

**Results:**

Metformin suppressed cell growth of 9 kinds of mesothelioma including immortalized cells of mesothelium origin irrespective of the p53 functional status, whereas susceptibility to nutlin-3a was partly dependent on the *p53* genotype. We investigated combinatory effects of metformin and nutlin-3a on, nutlin-3a sensitive MSTO-211H and NCI-H28 cells and insensitive EHMES-10 cells, all of which had the wild-type *p53* gene. Knockdown of p53 expression with the siRNA demonstrated that susceptibility of MSTO-211H and NCI-H28 cells to nutlin-3a was p53-dependent, whereas that of EHMES-10 cells was not. Nevertheless, all the cells treated with both agents produced additive or synergistic growth inhibitory effects. Cell cycle analyses also showed that the combination increased sub-G1 fractions greater than metformin or nutlin-3a alone in MSTO-211H and EHMES-10 cells. Western blot analyses showed that metformin inhibited downstream pathways of the mammalian target of rapamycin (mTOR) but did not activate the p53 pathways, whereas nutlin-3a phosphorylated p53 and suppressed mTOR pathways. Cleaved caspase-3 and conversion of LC3A/B were also detected but it was dependent on cells and treatments. The combination of both agents in MSTO-211H cells rather suppressed the p53 pathways that were activated by nutrin-3a treatments, whereas the combination rather augmented the p53 actions in NCI-H28 and EHMES-10 cells.

**Conclusion:**

These data collectively indicated a possible interactions between mTOR and p53 pathways, and the combinatory effects were attributable to differential mechanisms induced by a cross-talk between the pathways.

**Electronic supplementary material:**

The online version of this article (doi:10.1186/s12885-017-3300-y) contains supplementary material, which is available to authorized users.

## Background

Malignant mesothelioma, developed in the pleural cavity, is resistant to conventional treatments and the patient numbers are growing particularly in emerging countries [1]. A combination of cisplatin and pemetrexed, the current first-line chemotherapy, demonstrated its effectiveness compared with cisplatin alone [2], but no further improvement in the chemotherapy was reported for more than a decade. A possible second-line agent is not yet established and molecular-targeting agents turned out to be ineffective in current clinical trials [3].

Metformin, an agent for type 2 diabetes, showed the anti-tumor activity in various types of tumors, and the therapeutic effects were mainly attributable to inhibition of the mammalian target of rapamycin (mTOR) pathways through AMP-activated protein kinase (AMPK) and others molecules such as regulated in development and DNA damage responses 1 (REDD1) [4]. Many types of human tumors up-regulated expression of the mTOR complex 1 which regulated cell growth and metabolism according to their cellular energy levels, and suppression of the mTOR pathways inhibited tumor cell growth via 4E–BP1 and p70S6K molecules [5, 6]. Inhibition of the mTOR pathways is consequently one of the targeted areas for development of anti-cancer agents. An agent for suppressing the mTOR complex 1 activity, everolimus, was in fact demonstrated to inhibit tumor growth and is currently in use for renal cell carcinoma and breast cancer [7, 8]. An inhibitor for mTOR pathways in general suppressed cell cycle progression but the action mechanism was complex. Metformin, an inhibitor for mTOR pathways, showed a number of effects including induction of cycle arrest, apoptosis and autophagy, depending on the cell type tested [4, 5]. Previous studies showed that the mTOR pathways were often activated in many of mesothelioma clinical specimens and the elevated expression was linked with poor prognosis of the patients [9–11]. Nevertheless, an effect of metformin has not yet been examined in mesothelioma.

A majority of the *p53* genotype of clinical specimens from mesothelioma patients is wild-type but the INK4A/ARF region, which includes the *p14* and *p16* genes, is often deleted in the specimens [12]. The p14 defect in mesothelioma facilitated ubiquitin-mediated p53 degradation since p14 blocked a MDM2 action which degraded p53 through the ubiquitination-proteasome pathway. The genetic characteristic led to a functional p53 deficiency and suppressed the downstream pathways despite the wild-type *p53* genotype. Nutlin-3a, an inhibitor for interaction between MDM2 and p53, suppressed MDM2-mediated p53 ubiquitination, and subsequently augmented p53 expression levels by increasing p53 stability without any genotoxic stimulations [13]. Tumor cells bearing the wild-type *p53* gene in fact showed cell cycle arrest followed by apoptosis with nutlin-3a treatments [14, 15]. An inhibitor for the MDM2-p53 interaction is therefore a therapeutic agent for mesothelioma since up-regulation of endogenous wild-type p53 levels restores the p53 functions and activates the downstream pathways. In contrast, deficiency of p16 augmented phosphorylation of pRb and induced uninhibited cell growth. Increased p53 levels also inhibited the pRb phosphorylation through induction of p21, one of the p53 target molecules [16]. Consequently, up-regulation of p53 is a therapeutic strategy for mesothelioma by enhancing the downstream pathways and inhibiting cell cycle progression.

Interactions between the p53 pathways and the AMPK/mTOR pathways are not well characterized and are influenced by a number of factors. Growth signals through the insulin-like growth factor-mTOR pathways are regulated by metabolic conditions, and a cross-talk between the two pathways caused by genotoxicity is subjected to a number of cellular stresses. Accumulating data also suggest that the activated AMPK phosphorylated p53 at serine 15 residue, a marker for p53 activation, partly through inhibition of the mTOR functions, and that the activated p53 pathways in turn inhibited the mTOR activities through AMPK under stress or non-stress conditions [17–19]. Moreover, mTOR inhibitors, metformin and rapamycin, enhanced cytotoxicity of anti-cancer agents in *p53*-mutated tumors but rather protected normal cells with the wild-type *p53* from the drug-induced cytotoxicity [20]. We thereby examined anti-tumor effects of metformin and non-genotoxic nutlin-3a, and possible combinatory effects on mesothelioma under no metabolic stress. We further investigated a possible mechanism of the combinatory effects in terms of interactions between up-regulation of p53 levels and inhibition of the mTOR pathways.

## Methods

### Cells and agents

Human mesothelioma cells, MSTO-211H (CRL-2081), NCI-H28 (CRL-5820), NCI-H226 (CRL-5826), NCI-H2052 (CRL-5915) and NCI-H2452 (CRL-5946), and mesothelial cells immortalized with SV40 T antigen, Met-5A (CRL-9444), were purchased from American Type Culture Collection (Manassas, VA, USA), and JMN-1B, EHMES-1 and EHMES-10 cells were kindly provided by Dr. Hironobu Hamada, Hiroshima University, Japan [21]. The *p53* genotypes of JMN-1B and EHMES-1 cells are mutated and those of the others including Met-5A are wild-type. All the mesothelioma cells with the wild-type *p53* except Met-5A showed defective p14^ARF^ and p16^INK4A^ expression due to either lack of the transcription or deletion of the corresponding genomic DNA [12], whereas Met-5A cells had the *p14* ^*ARF*^ and *p16* ^*INKA*^ genes but lost the p53 functions because of SV40 T antigen expressed [22]. The *p53* genotype of NCI-H2452 was wild-type but p53 protein was truncated [23]. All the cells were cultured with RPMI 1640 supplemented with 10% fetal calf serum. Metformin (N, N-dimethylimidodicarbonimidic diamide hydrochloride) and nutlin-3a were purchased from Wako (Osaka, Japan) and Selleck Chemicals (Houston, TX, USA), respectively.

### In vitro cytotoxicity and cell counts

Cells (5 × 10^3^/well) were seeded in 96-well plates and were cultured for 4 days with different concentrations of an agent. Cell viability was determined with a cell-counting WST kit (Wako). The amount of formazan produced was determined with the absorbance at 450 nm and the relative viability was calculated based on the absorbance without any treatments. Cell numbers were also counted with the trypan blue dye exclusion assay. Combinatory effects were examined with CalcuSyn software (Biosoft, Cambridge, UK). Combination index (CI) values at respective fractions affected (Fa) points which showed relative levels of suppressed cell viability, were calculated based on the WST assay. CI < 1, CI = 1 and CI > 1 indicate synergistic, additive and antagonistic actions, respectively. Half maximal inhibitory concentration (IC_50_) values were also estimated with the CalcuSyn software.

### RNA interference

Cells were transfected with small interfering RNA (siRNA) duplex targeting p53 or with non-coding siRNA as a control (Invitrogen, Carlsbad, CA, USA) for 24 h using Lipofectamine RNAiMAX according to the manufacturer’s protocol (Invitrogen).

### Cell cycle analysis

Cells were treated with an agent were fixed in ice-cold 70% ethanol, incubated with RNase (50 μg/ml) and stained with propidium iodide (50 μg/ml). The staining profiles were analyzed with FACSCalibur (BD Biosciences, San Jose, CA, USA) and CellQuest software (BD Biosciences).

### Western blot analysis

Cell lysate was subjected to sodium dodecyl sulfate polyacrylamide gel electrophoresis. The protein was transferred to a nylon filter and was hybridized with antibody against AMPK (catalog number: #2532), phosphorylated AMPKα (Thr172) (#2535), 4E–BP1 (#9452), phosphorylated 4E–BP1 (#9459), p70 S6 kinase (p70S6K) (#9202), phosphorylated p70S6K (Thr389) (#9205), Bcl-2 (#2872), Bax (#2772), phosphorylated p53 (Ser15) (#9284), caspase-3 (#9668), cleaved caspase-3 (#9661), LC3A/B (#4108), Atg-5 (#2630), Beclin-1 (#3495) (Cell Signaling, Danvers, MA, USA), REDD1 (10638–1-AP) (Proteintech, Chicago, IL, USA), p53 (Ab-6, Clone DO-1) (Thermo Fisher Scientific, Fremont, CA, USA) and glyceraldehyde-3-phosphate dehydrogenase (GAPDH) (ab9484) (Abcam, Cambrige, UK) as a loading control followed by appropriate second antibody. Dimethyl sulfoxide (DMSO), a solvent for nutlin-3a, was also used as a control. The membranes were developed with the ECL system (GE Healthcare, Buckinghamshire, UK).

## Results

### Growth inhibitory effects of metformin or nutlin-3a on mesothelioma

**Fig. 1:**
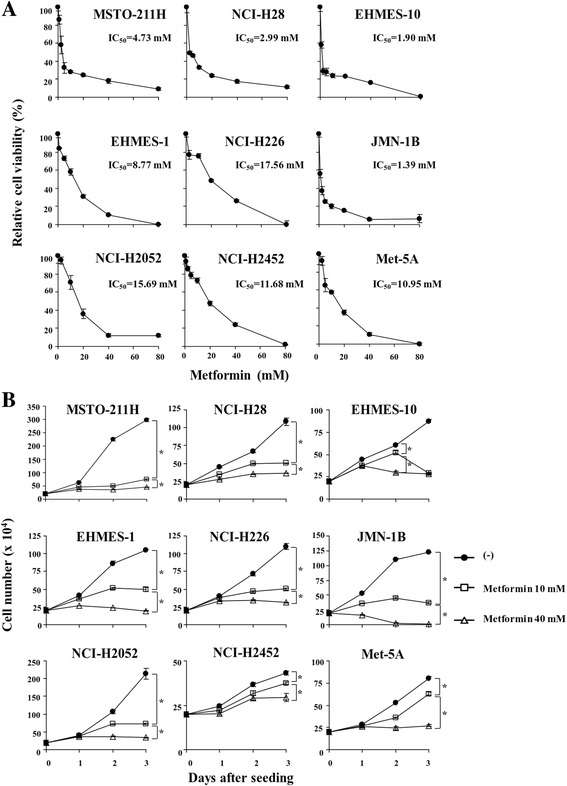
Susceptibility of mesothelioma and immortalized mesothelial cells to metformin. **a** Cells were treated with metformin at various concentrations for 4 days and the cell viabilities were measured with the WST assay. Relative viability was calculated based on untreated cells. IC_50_ values were calculated with CalcuSyn software. **b** Cells were treated with metformin as indicated and the live cell numbers were counted with a trypan *blue* dye exclusion assay. Averages and SE bars are shown (*n*=3). *P<0.05

We examined anti-tumor effects of metformin with the WST assay on 8 kinds of mesothelioma cells and an immortalized line, Met-5A cells, and compared the sensitivity with IC_50_ values according to the p53 functional status (Fig. 1a, Additional file 1: Table S1). EHMES-1 and JMN-1B cells with mutated *p53* gene, NCI-H2452 cells with truncated p53 protein that cannot induce p21 [23], and Met-5A cells with a loss of p53 functions by expressed SV40 T antigen that inactivated p53, were consequently classified as a non-functional p53 group and the others as a functional p53 group. These cells with the functional p53 in fact increased p53 responding to DNA damaging agents (data not shown). Metformin suppressed viability of all the cells but the relative viability was different among the cells tested. The susceptibility to metformin was independent of the p53 functionality. Average IC_50_ values of cells in the functional p53 group was 8.5 + 7.4 (SE) mM and that of cells in the non-functional p53 group was 8.2 + 3.5 mM (*P* = 0.93). We also tested growth of cells treated with metformin with a dye exclusion test (Fig. 1b). The suppressed growth rates varied among the cells but the proliferation was inhibited in a dose-dependent manner.

**Fig. 2:**
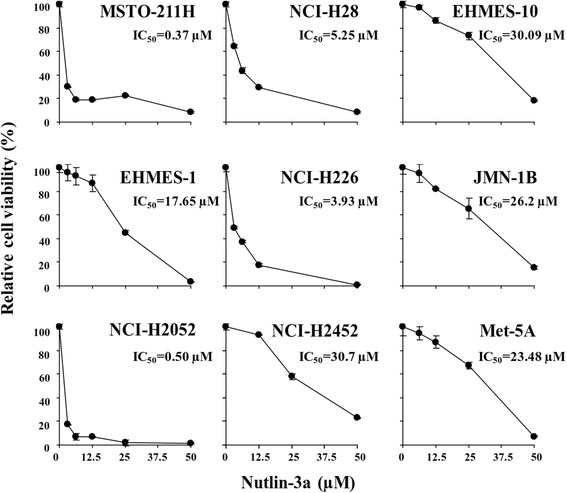
Susceptibility of mesothelioma and immortalized mesothelial cells to nutlin-3a. Cells were treated with nutlin-3a at various concentrations and the cell viabilities were measured with the WST assay. Relative viability was calculated based on uninfected cells. IC_50_ values were calculated with CalcuSyn software. Averages and SE bars are shown (*n*=3)

We investigated inhibitory effects of nutlin-3a with the WST assay on the mesothelioma cell panel (Fig. 2, Additional file 1: Table S1). Nutlin-3a blocked the interaction between p53 and MDM2, and consequently increased levels of p53, phosphorylated p53 and MDM2, one of the p53 target proteins, in mesothelioma with the wild-type *p53* gene (Additional file 2: Figure S1). The relative viability demonstrated that cells with functional p53 except EHMES-10 were susceptible to a low concentration of nutlin-3a (IC_50_; less than 6 μM), whereas others with non-functional p53 were relatively insensitive (IC_50_; more than 17 μM) (Fig. 2). Average IC_50_ values were lower in the functional p53 cells even including EHMES-10 cells (8.0 + 5.6 μM) than in the non-functional p53 cells (24.5 + 2.7) (*P* < 0.05). These data indicated that nutlin-3a suppressed viability of cells with intact p53 downstream pathways although EHMES-10 cells were less sensitive to nutlin-3a despite the wild-type *p53* gene.

### Combinatory effects of metformin and nutlin-3a

**Fig. 3:**
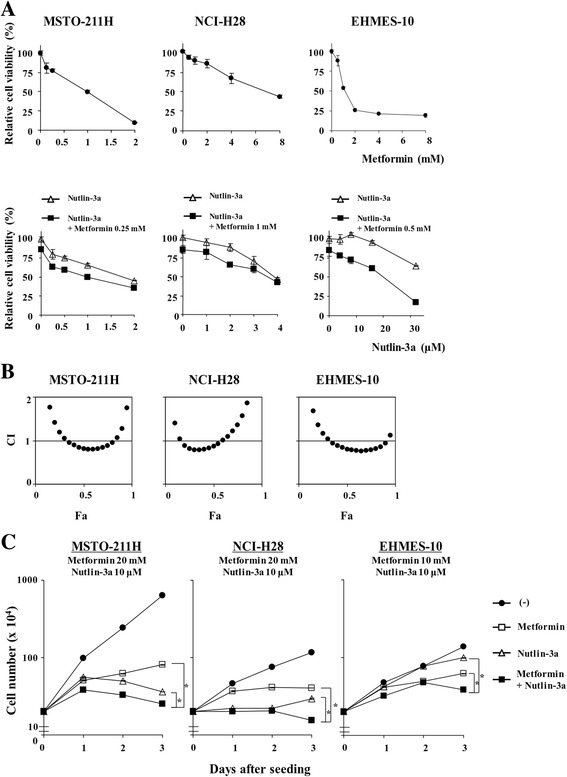
Combinatory effects of metformin and nutlin-3a. **a** Cells were treated with metformin, nutlin-3a or metformin plus nutlin-3a as indicated. Relative viability was calculated based on uninfected cells. Averages and SE bars are shown (*n*=3). **b** CI values in combination of metformin and nutlin-3a were calculated with CalcuSyn software at various Fa points. **c** Cell numbers were counted with a trypan blue dye exclusion assay after cells were treated with metformin, nutlin-3a or metformin plus nutlin-3a as indicated. Averages and SE bars are shown (*n*=3). *P<0.05

We selected 3 representative mesothelioma cells bearing the wild-type *p53* gene, MSTO-211H, NCI-H28 and EHMES-10 cells, to examine possible combinatory effects of metformin and nutlin-3a (Fig. 3a). All the cells were sensitive to metformin, while MSTO-211H and NCI-H28 but not EHMES-10 cells were sensitive to nutlin-3a. These data suggested that inhibition of mTOR pathways and activation of the p53 downstream pathways differentially produced cytotoxicity. We tested growth inhibitory actions with a low concentration of metformin and various concentrations of nutlin-3a. Analyses with the CalcuSyn software showed that combination of metformin and nutlin-3a produced additive or synergistic growth suppressive effects at Fa points between 0.35 and 0.8 in MSTO-211H and EHMES-10 cells, and between 0.2 and 0.6 in NCI-H28 cells (Fig. 3b). We also examined growth kinetics by the combination (Fig. 3c). We tested the growth retardation with a high metformin concentration to ensure the growth suppression and with nutlin-3a at 10 μM, which was enough to suppress growth of MSTO-211H and NCI-H28 cells but not EHMES-10 cells. Growth inhibition by nutlin-3a was subsequently minimum in EHMES-10 cells, but a combinatory use of metformin and nutlin-3a induced growth suppression greater than a single agent in all the cells including EHMES-10 cells. These data indicated that both agents produced combinatory effects.

### Involvement of p53 in metformin- and nutlin-3a-mediated effects

**Fig. 4:**
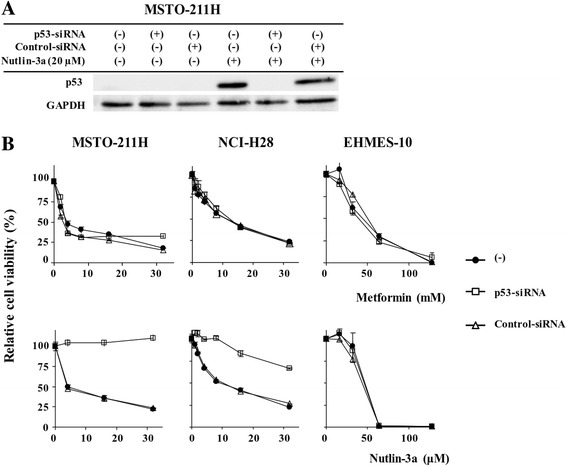
Involvement of p53 in metformin- and nutlin-3a-mediated cytotoxicity. **a** Western blot analysis to analyze p53 down-regulation. MSTO-211H cells treated with either p53-siRNA or control siRNA were incubated with nutlin-2a for 24 h. GAPDH expression was used as a loading control. **b** Cells were treated with p53-siRNA or control siRNA, and susceptibility to metformin or nutlin-3a was examined with the WST assay. Relative viability was calculated based on untreated cells. Averages and SE bars are shown (*n*=3)

**Fig. 5:**
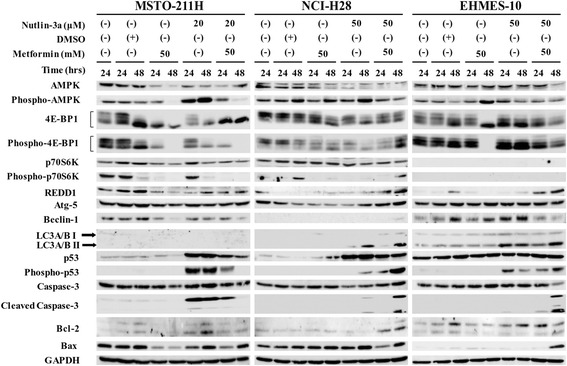
Western blot analyses with cells treated with metformin and/or nutlin-3a. Cells were treated with metformin, nutlin-3a, DMSO as a solvent control, or metformin pulse nutlin-3a at the indicated concentrations for 24 and 48 h. Cell lysates were probed with antibody as indicated. GAPDH was used as a loading control

We examined a role of p53 in metformin- and nutlin-3a-induced growth inhibition with cells treated with siRNA for p53 or control siRNA (Fig. 4). We firstly examined down-regulation of p53 expression in MSTO-211H cells treated with siRNA and/or nutlin-3a (Fig. 4a). Expression of p53 was scarcely detectable in MSTO-211H cells but was induced in nutlin-3a-treated cells. Treatments with p53-siRNA suppressed nutlin-3a-mediated p53 expression completely, whereas a control siRNA minimally influenced the p53 expression. These data indicated that nutlin-3a augmented p53 expression and the expression was depleted with p53-siRNA. Effects of metformin or nutlin-3a were then examined under the siRNA-treated condition (Fig. 4b). Down-regulation of p53 did not influence the susceptibility of any of the cells to metformin, indicating that the metformin-induced growth suppression was independent of the p53 pathways. In contrast, cytotoxicity of nutlin-3a was significantly reduced in MSTO-211H and NCI-H28 cells treated with p53-siRNA but not with control siRNA. Susceptibility of p53-siRNA-treated EHMES-10 cells to nutlin-3a remained unchanged, indicating that the p53 pathways was irrelevant to the growth suppression. The p53-independent cytotoxicity was associated with insensitivity of EHMES-10 cells to nutlin-3a. We also examined effects of nutlin-3a on p53 phosphorylation, which was a marker of p53 activation (Fig. 5, Additional file 2: Figure S1). Phosphorylation of p53 was induced in EHMES-10 cells as well as in MSTO-211H and NCI-H28 cells, indicating that the p53 pathways were also activated in EHMES-10 cells. These data therefore showed that growth inhibitory effects produced by nutlin-3a in EHMES-10 cells were attributable to a p53-independent mechanism.

### Cell cycle changes induced by metformin and nutlin-3a

We examined cell cycle progression of MSTO-211H, NCI-H28 and EHMES-10 cells after treatments of metformin, nutlin-3a or metformin plus nutlin-3a. We investigated the effects with different concentrations, metformin at 20 and 60 mM, and nutlin-3a at 10, 40 and 60 μM depending on cells (Table 1, Additional file 3: Figure S2). The cell cycle analyses showed that metformin at 20 mM did not influence cell cycles although viability with the WST assay was suppressed at the concentration. Metformin at 60 mM induced differential effects on cell cycle progression patterns. MSTO-211H cells showed increased sub-G1 fractions, whereas NCI-H28 cells and to a lesser extent EHMES-10 cells increased G2/M populations. Nutlin-3a also showed differential effects on cell cycle progressions. MSTO-211H cells, sensitive to nutlin-3a, were tested at 10 μM, and NCI-H28 cells were also treated at 40 μM. MSTO-211H cells increased G1 and decreased S-phase fractions, whereas NCI-H28 cells increased G2/M populations at both 10 and 40 μM. Augmented sub-G1 fractions followed thereafter in both cells. Cell cycle patterns of EHMES-10 cells were not influenced at 10 μM since the cells were insensitive to the dose of nutlin-3a, but showed increased G1 with decreased S-phase populations at 60 μM.

**Table 1:** Cell cycle changes caused by metformin and/or nutlin-3a

Cells	Time (hrs)	Treatment	Cell cycle distribution (%)(Average ± SE)			
			Sub-G1	G1	S	G2/M
MSTO-211H	24	(-)	4.35 ± 0.22	56.30 ± 0.35	17.93 ± 0.04	20.88 ± 0.44
		Met 20mM	5.43 ± 0.23	68.82 ± 0.25	12.91 ± 0.22	12.39 ± 0.23
		Met 60 mM	10.92 ± 0.63	63.25 ± 1.75	4.71 ± 0.16	20.88 ± 2.13
		Nut 10 μM	5.95 ± 0.11	84.18 ± 0.43	2.35 ± 0.16	7.32 ± 0.23
		Met 20mM + Nut 10 μM	7.39 ± 0.14	72.91 ± 0.29	3.00 ± 0.14	16.23 ± 0.29
		Met 60 mM + Nut 10 μM	10.56 ± 1.42	61.41 ± 1.50	7.93 ± 0.69	19.71 ± 0.71
	48	(-)	0.76 ± 0.03	68.87 ± 0.65	12.70 ± 0.59	17.31 ± 0.20
		Met 20mM	3.40 ± 0.06	70.64 ± 0.26	11.96 ± 0.06	13.43 ± 0.21
		Met 60 mM	7.44 ± 0.12*	68.10 ± 0.30	5.29 ± 0.08	18.84 ± 0.13
		Nut 10 μM	6.11 ± 0.22*	85.90 ± 0.25	1.89 ± 0.03	5.92 ± 0.13
		Met 20mM + Nut 10 μM	6.51 ± 0.16	76.44 ± 0.24	2.82 ± 0.08	13.87 ± 0.18
		Met 60 mM + Nut 10 μM	11.34 ± 0.22*	63.25 ± 0.24	2.97 ± 0.04	22.25 ± 0.22
	72	(-)	1.15 ± 0.11	82.29 ± 0.39	4.04 ± 0.17	12.26 ± 0.18
		Met 20mM	3.08 ± 0.09	76.19 ± 0.21	8.07 ± 0.14	12.25 ± 0.42
		Met 60 mM	13.28 ± 0.27*	65.29 ± 1.07	4.99 ± 0.10	16.24 ± 0.71
		Nut 10 μM	11.38 ± 0.14*	81.30 ± 0.28	1.88 ± 0.10	5.29 ± 0.13
		Met 20mM + Nut 10 μM	7.39 ± 0.15	75.38 ± 0.21	2.63 ± 0.06	14.26 ± 0.23
		Met 60 mM + Nut 10 μM	36.25 ± 0.44*	44.02 ± 0.25	3.28 ± 0.06	16.15 ± 0.26
NCI-H28	24	(-)	0.47 ± 0.04	60.27 ± 0.19	16.66 ± 0.34	22.06 ± 0.25
		Met 20mM	0.43 ± 0.02	67.39 ± 0.41	11.76 ± 0.29	19.66 ± 0.13
		Met 60 mM	0.89 ± 0.08	44.81 ± 0.07	15.01 ± 0.17	38.33 ± 0.17
		Nut 10 μM	0.77 ± 0.02	40.63 ± 0.20	9.25 ± 0.42	47.61 ± 0.36
		Nut 40 μM	1.22 ± 0.07	44.87 ± 0.57	15.17 ± 0.57	37.12 ± 0.65
		Met 20mM + Nut 10 μM	1.25 ± 0.04	42.33 ± 0.36	13.30 ± 0.38	41.28 ± 0.06
		Met 60 mM + Nut 40 μM	1.24 ± 0.15	48.20 ± 0.37	20.92 ± 0.17	28.35 ± 0.38
	48	(-)	1.54 ± 0.05	60.08 ± 0.39	16.04 ± 0.36	21.88 ± 0.01
		Met 20mM	0.45 ± 0.04	73.97 ± 0.18	6.35 ± 0.05	18.66 ± 0.10
		Met 60 mM	1.06 ± 0.10	40.68 ± 0.08	14.65 ± 0.19	42.61 ± 0.19
		Nut 10 μM	3.84 ± 0.10	43.82 ± 0.29	8.28 ± 0.20	42.47 ± 0.26
		Nut 40 μM	2.50 ± 0.10	49.77 ± 0.22	10.35 ± 0.10	36.08 ± 0.21
		Met 20mM + Nut 10 μM	0.91 ± 0.09	46.45 ± 0.74	9.23 ± 0.29	41.90 ± 0.90
		Met 60 mM + Nut 40 μM	1.38 ± 0.12	49.24 ± 0.21	20.16 ± 0.30	27.63 ± 0.15
	72	(-)	1.49 ± 0.02	64.68 ± 0.34	15.58 ± 0.03	17.79 ± 0.30
		Met 20mM	0.52 ± 0.09	75.59 ± 0.14	4.52 ± 0.08	18.86 ± 0.21
		Met 60 mM	1.50 ± 0.31	41.66 ± 0.37	15.31 ± 0.03	40.59 ± 0.27
		Nut 10 μM	6.09 ± 0.20	43.98 ± 0.25	8.75 ± 0.23	39.57 ± 0.41
		Nut 40 μM	8.96 ± 0.83	61.92 ± 0.79	7.31 ± 0.02	21.05 ± 0.49
		Met 20mM + Nut 10 μM	1.01 ± 0.28	46.14 ± 0.66	9.14 ± 0.11	42.20 ± 0.41
		Met 60 mM + Nut 40 μM	9.00 ± 0.72	44.61 ± 0.43	15.83 ± 0.25	29.48 ± 0.45
EHMES-10	24	(-)	0.46 ± 0.05	70.86 ± 0.69	9.64 ± 0.3	18.63 ± 0.57
		Met 20mM	1.04 ± 0.06	73.58 ± 0.31	8.78 ± 0.05	16.18 ± 0.32
		Met 60 mM	1.61 ± 0.06	67.93 ± 0.31	6.95 ± 0.14	23.13 ± 0.28
		Nut 20 μM	1.19 ± 0.10	73.47 ± 0.21	10.38 ± 0.26	14.38 ± 0.29
		Nut 60 μM	1.19 ± 0.08	80.17 ± 0.16	3.49 ± 0.06	14.74 ± 0.05
		Met 20mM + Nut 20 μM	0.85 ± 0.02	76.79 ± 0.23	7.35 ± 0.09	14.61 ± 0.19
		Met 60 mM + Nut 60 μM	2.37 ± 0.19	73.06 ± 0.38	5.2 ± 0.38	18.93 ± 0.32
	48	(-)	0.92 ± 0.04	73.14 ± 0.06	10.34 ± 0.23	15.22 ± 0.35
		Met 20mM	1.46 ± 0.13	61.87 ± 0.16	13.00 ± 0.12	23.04 ± 0.21
		Met 60 mM	2.67 ± 0.12*	66.72 ± 0.12	9.32 ± 0.11	20.73 ± 0.13
		Nut 20 μM	1.62 ± 0.20	67.78 ± 0.21	12.12 ± 0.17	18.02 ± 0.17
		Nut 60 μM	1.85 ± 0.16*	84.44 ± 0.12	3.32 ± 0.05	10.15 ± 0.06
		Met 20mM + Nut 20 μM	1.35 ± 0.04	66.71 ± 0.35	13.01 ± 0.32	18.19 ± 0.42
		Met 60 mM + Nut 60 μM	4.57 ± 0.17*	74.63 ± 0.17	5.44 ± 0.16	15.11 ± 0.2
	72	(-)	2.18 ± 0.55	56.86 ± 0.62	12.2 ± 0.75	27.79 ± 1.57
		Met 20mM	1.89 ± 0.11	63.77 ± 0.19	11.90 ± 0.21	21.64 ± 0.42
		Met 60 mM	3.07 ± 0.14*	64.22 ± 0.13	8.22 ± 0.12	23.99 ± 0.22
		Nut 20 μM	0.73 ± 0.03	67.46 ± 0.28	11.05 ± 0.19	20.22 ± 0.39
		Nut 60 μM	3.47 ± 0.11*	84.27 ± 0.25	3.4 ± 0.17	8.74 ± 0.07
		Met 20mM + Nut 20 μM	1.27 ± 0.03	70.85 ± 0.23	9.44 ± 0.43	17.85 ± 0.19
		Met 60 mM + Nut 60 μM	13.85 ± 0.86*	62.12 ± 0.26	6.35 ± 0.25	17.57 ± 0.86

*P < 0.05, compared between combination and either metformin or nutlin-3a single treatment. *N* = 3

*Met* Metformin, *Nut* Nutlin-3a

A combinatory use of metformin at 20 mM and nutlin-3a at 10 μM did not influence the cell cycle in any of the cells. We therefore increased metformin concentration at 60 mM. Combination of metformin and nutlin-3a at 10 μM increased sub-G1 populations in MSTO-211H cells, and that of metformin with nutlin-3a at 60 μM also augmented sub-G1 fractions in EHMES-10 cells. The increased sub-G1 fraction was greater than that caused by single agent alone. In contrast, cell cycle changes by the combination with 40 μM of nutlin-3a in NCI-H28 cells remained the same as those by nutlin-3a alone, and metformin did not influence cell cycle patterns in the combination. Cell cycle changes were thus differentially induced in the cells tested. MSTO-211H cells were prone to be arrested at G1 phase followed by increased sub-G1 populations, while NCI-H28 cells were likely to be arrested at G2/M phase. In contrast, EHMES-10 cells showed complex results in cell cycle progressions.

### Differential influence of metformin and nutlin-3a on signal pathways

We investigated molecular events in cells treated with metformin, nutlin-3a or the combination and analyzed a possible involvement of the mTOR and the p53 downstream pathways (Fig. 5). The agent concentrations for Western blot analyses were similar to those used for cell cycle analyses except nutlin-3a at 20 μM in MSTO-211H cells because the concentration induced p53 expression in the cells and the induction was blocked by siRNA for p53 (Fig. 4a). Metformin treatments induced different responses on the AMPK/mTOR-mediated pathways. MSTO-211H cells treated with metformin down-regulated AMPK, 4E–BP1, REDD1 and to a lesser extent p70S6K levels, and subsequently phosphorylated AMPK, 4E–BP1 and p70S6K levels decreased. These data indicated that metformin suppressed the mTOR pathways in an AMPK- and a REDD1-independent manners. In contrast, NCI-H28 cells treated with metformin dephosphorylated 4E–BP1 and p70S6K despite unchanged AMPK phosphorylation levels, suggesting that metformin suppressed the mTOR pathways without augmenting the AMPK activity. EHMES-10 cells treated with metformin showed increased phosphorylated AMPK and down-regulated 4E–BP1 phosphorylation, indicating that suppression of the mTOR pathways was associated with AMPK activation. These data showed that metformin induced mTOR inhibition but involvement of AMPK was inconsistent among the cells. We further examined apoptosis and autophagy pathways in metformin-treated cells. MSTO-211H cells showed down-regulation of Bax, Atg-5 and Beclin-1 expression levels but cleavage of caspase-3 was not induced. NCI-H28 showed slight increase of Bax and decrease of Atg-5 without caspase-3 cleavage, and EHMES-10 cells did not show any changes in expressions of molecules associated with apoptosis or autophagy compared with solvent-treated cells as a control. Expression of p53 was minimally increased in NCI-H28 cells but remained unchanged in MSTO-211H and EHMES-10 cells. Moreover, phosphorylation of p53 was not induced in any of the cells. These analysis indicated that both apoptosis and autophagy did not play a major role in metformin-induced growth suppression and that inhibited mTOR pathways scarcely influenced p53 levels and the downstream.

Nutlin-3a augmented p53 and the phosphorylation levels in all the cells although the induction levels were different among the cells. Cleaved caspase-3 levels were induced in MSTO-211H and to a lesser extent in NCI-H28 cells but not in EHMES-10 cells. The differential cleavage may be linked with p53 induction levels. Bax expression was up-regulated only in NCI-H28 cells. Conversion from LC3A/B I to LC3A/B II was detected in nutlin-3a-treated NCI-H28 and EHMES-10 cells but not in MSTO-211H cells. Beclin-1 was minimally up-regulated in EHMES-10 cells but not in other cells, and up-regulated Atg-5 expression was not detected in all the cells. These data consequently indicated that nutlin-3a augmented apoptosis through p53 in MSTO-211H cells but enhanced autophagy in EHMES-10 cells. In contrast, NCI-H28 cells to a lesser extent showed activation of both apoptosis and autophagy. Nutlin-3a up-regulated AMPK phosphorylation and decreased phosphorylation levels of 4E–BP1 and p70S6K in MSTO-211H and to a lesser extent in NCI-H28 cells, whereas EHMES-10 cells did not show any changes in these phosphorylation levels. Expression of REDD-1 decreased in MSTO-211H cells at 24 h but the expression in NCI-211H and EHMES-10 cells was minimally changed. These data collectively showed that nutlin-3a augmented p53-mediated apoptosis in MESO-211H and NCI-H28 cells, and autophagy was also involved in NCI-H28 and EHMES-10 cells. Up-regulated p53 expression thus inhibited the mTOR pathways in nutlin-3a-sensitive cells.

Combination of metformin and nutlin-3a decreased apoptotic pathways in MSTO-211H cells. The combination decreased p53 and the phosphorylation levels and consequently cleavage caspase-3 levels were down-regulated. Expression of Bax, Bcl-2, Atg-5 and Bclin-1 levels in MSTO-211H cells were minimally changed and the expression levels were almost similar to those at between metformin- and nutlin-3a-treated cells. The combination down-regulated levels of AMPK, phosphorylated AMPK, phosphorylated 4E–BP-1, p70S6K and phosphorylated p70S6K and the levels were also comparable to those in metformin-treated cells. These data suggested that metformin suppressed nutlin-3a-mediated effects in MSTO-211H cells. In contrast, NCI-H28 cells showed further increase of p53 phosphorylation, cleaved caspase-3 and Bcl-2 levels with the combination, but the levels of Atg-5 and to a lesser degree LC3A/B increased compared with nutlin-3a-treated cells. The combination also augmented REDD1 expression and phosphorylation of 4E–BP1 and p70S6K, but down-regulated phosphorylation levels of AMPK, indicating that the mTOR pathways rather activated in NCI-H28 cells despite of enhanced apoptosis and autophagy signaling. EHMES-10 cells treated with the combination increased cleavage of caspas-3 and Bax but p53 phosphorylation levels remained unchanged. Beclin-1 expression levels decreased but a ratio between LC3A/B I and LC3A/B II was not different from that of nutlin-3a-treated cells. As for the mTOR pathways, the phosphorylated 4E–BP1 level in the combination was similar to that of between metformin-treated and nutlin-3a-treated cells. Furthermore, REDD1 expression increased in the combinatory treatments, and phosphorylated AMPK levels were slightly down-regulated in EHMES-10 cells. These data showed that the combination induced apoptosis without further p53 activation and suppressed the mTOR pathways through an augmented REDD1 level despite down-regulated AMPK actions. These molecular analyses collectively suggested that growth inhibition produced by the combination was attributable to multiple mechanisms including apoptosis, autophagy, and machinery irrelevant to apoptosis or autophagy. Metformin also influenced negatively or positively the p53 pathways, suppressed the pathways in MSTO-211H cells but augmented in NCI-H28 and EHMES-10 cells.

## Discussion

We showed in the present study that metformin suppressed growth of mesothelioma in a p53-independent manner and firstly reported to our knowledge that a combinatory use of metformin and nutlin-3a produced additive or synergistic inhibitory effects. The mechanism underlying the metformin- and combination-mediated growth suppression was complex and non-AMPK/mTOR pathways were also involved. Moreover, nutlin-3a-mediated augmentation of p53 inhibited the mTOR pathways but metformin did not influence p53 levels without nutlin-3a. Nevertheless, metformin affected p53 activation either positively or negatively under nutlin-3a treatments.

Metformin produced cytotoxic effects on human tumors and multiple mechanisms were involved in the anti-tumor activities [4]. We used mesothelioma and immortalized cells of a mesothelium origin, and examined the metformin-induced growth suppression in terms of the *p53* genotype. The suppressive activity was not associated with the *p53* status or with functionality of the p53 downstream pathways in contrast to nutlin-3a. Moreover, down-regulated p53 with siRNA but did not influence metformin-mediated suppression. Metformin scarcely modulated p53 and the phosphorylated p53, and apoptosis as well as autophagy were not involved in the growth inhibition. Cell cycle progression patterns under the non-apoptosis and non-autophagy conditions showed either increased sub-G1 or G2/M fractions depending on cells tested.

We showed that metformin-induced suppression of cell viability was attributable to multiple systems. Previous studies demonstrated at least 4 mechanisms responsible for the suppression, (A) activation of AMPK with down-regulated mTOR actions [4, 5], (B) suppression of the mTOR complex 1 activities through augmented REDD1-mediated pathways without an AMPK involvement [24], (C) inhibition of the Stat3/Bcl-2 signal [25], and (D) modulation of miRNAs which includes augmented miRNA let7A and down-regulated miRNA 181 [26]. Metformin-treated MSTO-211H cells showed decreased phosphorylation of mTOR downstream molecules without enhanced AMPK phosphorylation, suggesting the (B) mechanism with little involvement of apoptosis or autophagy-mediated pathways. Decreased AMPK phosphorylation did not however deny an involvement of AMPK in the metformin-mediated suppression. We in fact found that an AMPK inhibitor, compound C, negated the metformin effects, which indicated that AMPK activation played a role in the suppression (Additional file 4: Figure S3). Moreover, metformin also down-regulated REDD1 expression, which suggested that regulation of the mTOR pathways was balanced between AMPK and REDD1 pathways in MSTO-211H cells. In contrast, metformin-treated NCI-H28 cells decreased phosphorylation of the mTOR downstream molecules without AMPK or REDD1 activations, indicating mTOR regulations bypassing the AMPK and the REDD1 pathways. EHMES-10 cells treated with metformin inhibited mTOR pathways through AMPK activation as listed in the (A) mechanism. We detected little changes of Bcl-2 expression levels and the mechanism (C) was not involved in mesothelioma tested. These data collectively indicated that metformin suppressed mTOR downstream pathways with a differential involvement of AMPK and REDD1 molecules. A possible involvement of p53-mediated apoptosis was scarcely detected in all the cells tested and autophagy might minimally contribute to the growth inhibition in MSTO-211H cells. These data together with the p53-siRNA treatments indicated that metformin did not influence the p53 pathways and cell cycle changes induced by metformin were independent of the pathways.

A mechanism of nutlin-3a-mediated growth inhibition was less complex than that of metformin. Nutlin-3a phosphorylated p53 in all the cells tested, and induced apoptosis at different levels and to some extent autophagy. Moreover, augmentation of p53 by nutlin-3a induced inhibition of the mTOR pathways, which was evidenced by dephosphorylation of 4E–BP1 and p70S6K. In contrast, EHMES-10 cells were susceptible to nutlin-3a only at a high concentration, and induced the LC3A/B conversion without caspase-3 cleavages under the condition. These data suggested a possible involvement of autophagy as an off-target effect of nutlin-3a. In addition, EHMES-10 cells did not improve sensitivity to nutlin-3a in a p53-siRNA treatment. Interestingly, a recent study demonstrated that nutlin-3a induced autophagy in *p53* wild-type cells, and activation of AMPK was involved in the cell death [27]. We observed the same mechanism operating in nutlin-3a-treated NCI-H28 cells. EHMES-10 cells however did not show any changes in the AMPK/mTOR systems, which further suggested an AMPK-independent autophagy induced by nutlin-3a.

Combination of metformin and nutlin-3a produced additive or synergistic effects detected with the WST and the dye exclusion test. MSTO-211H cells treated with both agents showed enhanced sub-G1 fractions but the mechanism underlying the cytotoxicity, in the context of the AMPK/mTOR pathways and the p53 activation, seemed to be similar to that in the metformin-treated case. Nutlin-3a alone augmented mTOR inhibition in MSTO-211H cells but the combination with metformin did not further suppress the mTOR pathways. Furthermore, metformin rather down-regulated nutlin-3a-induced effects. These data collectively indicated that increased cell death in the combination was not associated with apoptosis or inhibited mTOR pathways. The combinatory effects were therefore not due to just augmentation of the metformin-induced signal pathways, but an additional undefined mechanism should be involved. NCI-H28 and EHMES-10 cells augmented caspase-3 cleavage levels in the combination. In contrast, cell cycle analyses showed increased sub-G1 populations in EHMES-10 cells but not in NCI-H28 cells. NCI-H28 cells treated with both agents increased conversion of LC3A/B which was accompanied by autophagy, and augmented Bcl-2 expression which might block apoptosis. These data suggested that NCI-H28 cells were subjected to autophagy rather than apoptosis. Metformin in fact decreased Atg-5 levels but the combination with nutlin-3a restored the levels. We also found that the down-regulated Atg-5 was irrelevant to AMPK activation since an AMPK activator, A769662, did not influence the Atg-5 expression (Additional file 5: Figure S4A). In contrast, EHMES-10 cells treated with the combination did not show further conversion of LC3A/B or Bcl-2 up-regulation but augmented Bax expression, which indicated that EHMES-10 cells were prone to be apoptotic. The dissimilar ratios of sub-G1 fraction between NCI-H28 and EHMES-10 cells can therefore be attributable to the differential cell death mechanisms. Influence on the AMPK/mTOR pathways could also bring the dissimilar results between NCI-H28 and EHMES-10 cells. Both cells augmented REDD1 expression levels and down-regulated phosphorylated AMPK levels in the combination, but phosphorylated 4E–BP1 and p70S6K levels, mapped in the mTOR pathways, were rather up-regulated in NCI-H28 cells. In contrast, EHMES-10 cells did not show such up-regulated mTOR pathways. These data collectively suggested that the combinatory effects was attributable to enhanced apoptotic and/or non-apoptotic pathways, and contribution of the mTOR pathways to the effects was inconsistent. We also found that AMPK activation itself might not directly contribute to the combinatory effects with nutlin-3a in NCI-H28 cells (Additional file 5: Figure S4B, Additional file 6: Table S2), which could be associated with up-regulated mTOR downstream pathways and further indicated complexity of mechanism regarding the combinatory effects.

Mesothelioma has another frequent genetic alternations, mutations of *NF2* and those of the downstream genes found in about 50% of the clinical specimens [28]. The loss of functions caused by these mutation led to activation of the Hippo pathway, and the activation augmented the mTOR downstream pathways [29]. A combinatory use of metformin and nutlin-3a in mesothelioma therefore affects a possible cross-talk between the p53 and the Hippo pathways. The present analyses of the AMPK pathways after nutrin-3a treatments in the *p53* wild-type cells revealed the cross-talk under no metabolic and genotoxic stresses. We showed that nutlin-3a-mediated p53 up-regulation activated the AMPK pathways and inhibited the mTOR pathways in MSTO-211 and NCI-H28 cells, whereas activation of AMPK did not directly enhance p53 levels in NCI-H28 cells (Additional file 5: Figure S4A). On the other hand, EHMES-10 cells treated with nutlin-3a did not activate the AMPK and failed to cleave caspase-3. Furthermore, metformin negated nutlin-3a-induced activation of p53 and the AMPK pathways, and induced non-apoptotic pathways in MSTO-211H cells. The combinatory effects in NCI-H28 and EHMES-10 cells were however primarily produced by enhanced apoptosis. These data indicated that metformin produced bivalent actions on the p53 pathways, inhibition as observed in MSTO-211H cells and augmentation in NCI-H28 and EHMES-10 cells. Recently, Cho et al. showed that decreased NF2 expression further down-regulated p53 levels through Snail in mesothelioma, and an inhibitor for Snail restored the p53 levels [30]. Combination of an mTOR inhibitor and an agent to augment p53 levels is therefore a suitable therapeutic strategy for mesothelioma with the *NF2* mutation and loss of p53 functions. Previous studies also showed that metformin up-regulated p53 levels through inhibited mTOR pathways and augmentation of p53 suppressed the mTOR activity [31]. The present study demonstrated that metformin did not influence p53 levels but activated the p53 pathways in combination with nutlin-3a. Furthermore, we showed that p53 up-regulation by nutlin-3a inhibited mTOR pathways although these effects were dependent on cells tested. The current study indicated a possible cross-talk between the p53 activation and the AMPK/mTOR pathways. Nevertheless, the cross-talk did not produce consistent outcomes as demonstrated in MSTO-211H cells, which could be attributable to differential genetic backgrounds among tumor cells used. Variable expression levels of respective molecules in the p53 and AMPK/mTOR pathways and their differential functional roles in the cell death can also contribute to the divergent outcomes.

## Conclusions

In conclusions, we examined cytotoxicity of metformin and nutlin-3a with a panel of mesothelioma and demonstrated that both agents produced combinatory effects on cell growth. A mechanism of the combination was however different among the cells tested probably due to heterogeneity of mesothelioma. Nevertheless, the present study suggest that a combinatory use of an inhibitor for mTOR and a p53-activating agent targets mesothelioma with characteristic genetic alterations and is a new therapeutic regimen. Metformin is now clinically in use and some of the MDM2-p53 inhibitors are under clinical trials, which indicates feasibility of the combination in mesothelioma treatments.

## Additional files

Additional file 1: Table S1. Sensitivity of mesothelioma cells to agents. (DOCX 15 kb)

Additional file 2: Figure S1. Expression of p53 and MDM2 in mesothelioma cells treated with nutlin-3a. Cells were treated with nutlin-3a as indicated and were probed with antibody against p53 (Ab-6, Clone DO-1) (Thermo Fisher Scientific), phosphorylated p53 (Ser 15) (#9284) (Cell Signaling), MDM2 (sc-965) (Santa Cruz Biotechnology) and GAPDH (ab9484) (Abcam) as a loading control. (PDF 125 kb)

Additional file 3: Figure S2. Cell cycle changes caused by metformin and/or nutlin-3a. Cells were treated with metformin and nutlin-3a as indicated, and analyzed for the cell cycle with a flow cytometry. (PDF 138 kb)

Additional file 4: Figure S3. AMPK inhibition blocked metformin-mediated suppression. MSTO-211H cells were treated with metformin and compound C as indicated for 4 days and relative cell viability was examined with the WST assay. Viability of cells treated with metformin at 5 mM but without compound C (A) and that without metformin or compound C (B) were shown as 100%. Averages and SEs are shown (*n* = 3). (PDF 72 kb)

Additional file 5: Figure S4. (A) AMPK activation was irrelevant to Atg-5 and p53 expression. NCI-H28 cells treated with A769662, an AMPK activator, did not influence Atg-5, p53 or phosphorylated p53 levels. (B) An AMPK activator did not produce synergistic combinatory effects with nutlin-3a. NCI-H28 cells were treated with A769662 and nutlin-3a as indicated for 4 days and relative cell viability was examined with the WST assay. Averages and SEs are shown (*n* = 3). (PDF 106 kb)

Additional file 6: Table S2. Combination Index by nutlin-3a and A769662. (DOCX 14 kb)

## Abbreviations

AMPK: AMP-activated protein kinase
CI: Combination index
DMSO: Dimethyl sulfoxide
Fa: Fractions affected
GAPDH: Glyceraldehyde-3-phosphate dehydrogenase
IC_50_: Half maximal inhibitory concentration
mTOR: Mammalian target of rapamycin
REDD1: Regulated in development and DNA damage responses 1
siRNA: Small interfering RNA
