# Supplementary material for: Metformin produces growth inhibitory effects in combination with nutlin-3a on malignant mesothelioma through a cross-talk between mTOR and p53 pathways
Source: BMC Cancer. 2017 May 2;17:309. doi: 10.1186/s12885-017-3300-y (PMC5414226; doi:10.1186/s12885-017-3300-y)
Supplement: Supplementary file 1 — Sensitivity of mesothelioma cells to agents. (DOCX 15 kb) [file 12885_2017_3300_MOESM1_ESM.docx]

Table S1. Sensitivity of mesothelioma cells to agents

Cells p53 genotype IC_50_ value (average + SE)

Metformin (mM) Nutlin-3a (μM)

MSTO-211H Wild-type 4.72 + 0.80 0.37 + 0.02

NCI-H28 Wild-type 2.99 + 0.30 5.25 + 0.19

EHMES-10 Wild-type 1.90 + 0.34 30.09 + 0.61

NCI-H226 Wild-type 17.56 + 2.22 3.93 + 0.12

NCI-H2052 Wild-type 15.69 + 2.52 0.50 + 0.33

EHMES-1 Mutated 8.77 + 0.24 17.65 + 0.82

JMN-1B Mutated 1.39 + 0.19 26.2 + 1.44

HCI-H2452 Wild-type 11.68 + 0.41 30.7 + 0.54

(truncated p53 protein)*

Met-5A Wild-type 10.95 + 1.06 23.48 + 4.01

(SV40 T expressed)*

*NCI-H2452 cells, expressing a truncated form of p53 protein, and Met-5A cells, transfected with the *SV40 T* antigen gene, were defective of the p53 functions. n=3.
