# Supplementary figures and images for: Metformin produces growth inhibitory effects in combination with nutlin-3a on malignant mesothelioma through a cross-talk between mTOR and p53 pathways
Source: BMC Cancer. 2017 May 2;17:309. doi: 10.1186/s12885-017-3300-y (PMC5414226; doi:10.1186/s12885-017-3300-y)

**Figure S1**

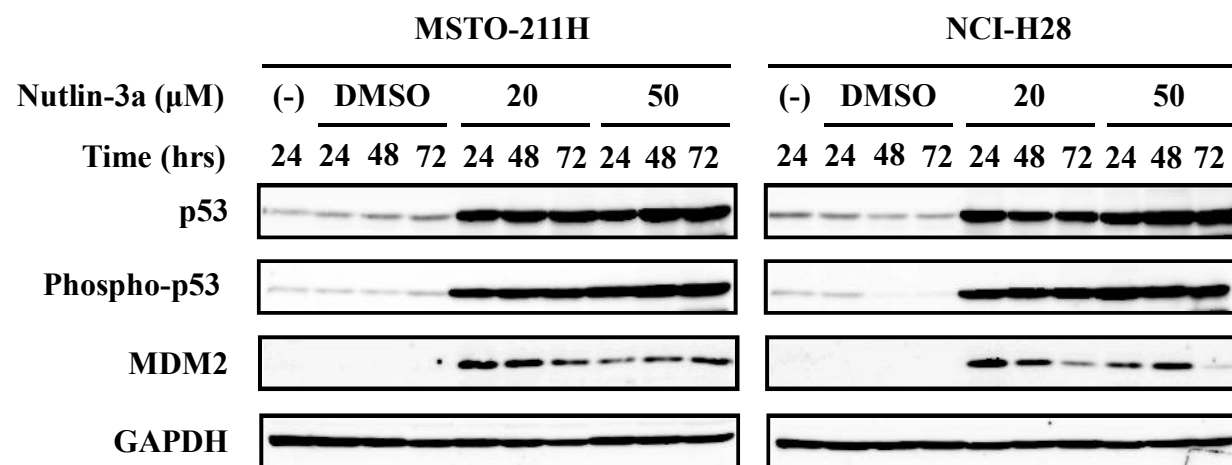

Supplement: Supplementary file 2 — Expression of p53 and MDM2 in mesothelioma cells treated with nutlin-3a. Cells were treated with nutlin-3a as indicated and were probed with antibody against p53 (Ab-6, Clone DO-1) (Thermo Fisher Scientific), phosphorylated p53 (Ser 15) (#9284) (Cell Signaling), MDM2 (sc-965) (Santa Cruz Biotechnology) and GAPDH (ab9484) (Abcam) as a loading control. (PDF 125 kb) [file 12885_2017_3300_MOESM2_ESM.pdf]

Figure S2

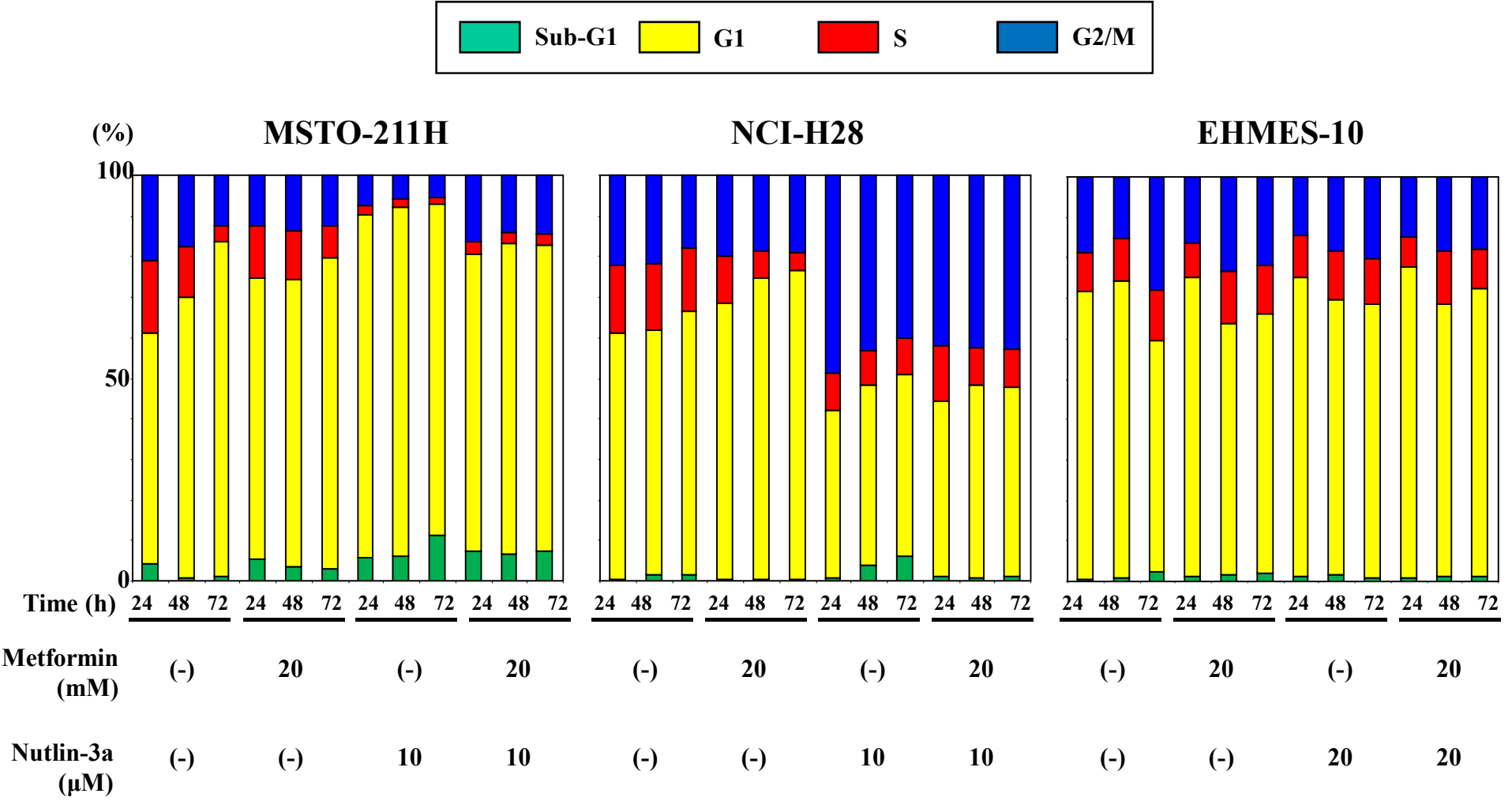

Supplementary Figure 2 (continued)

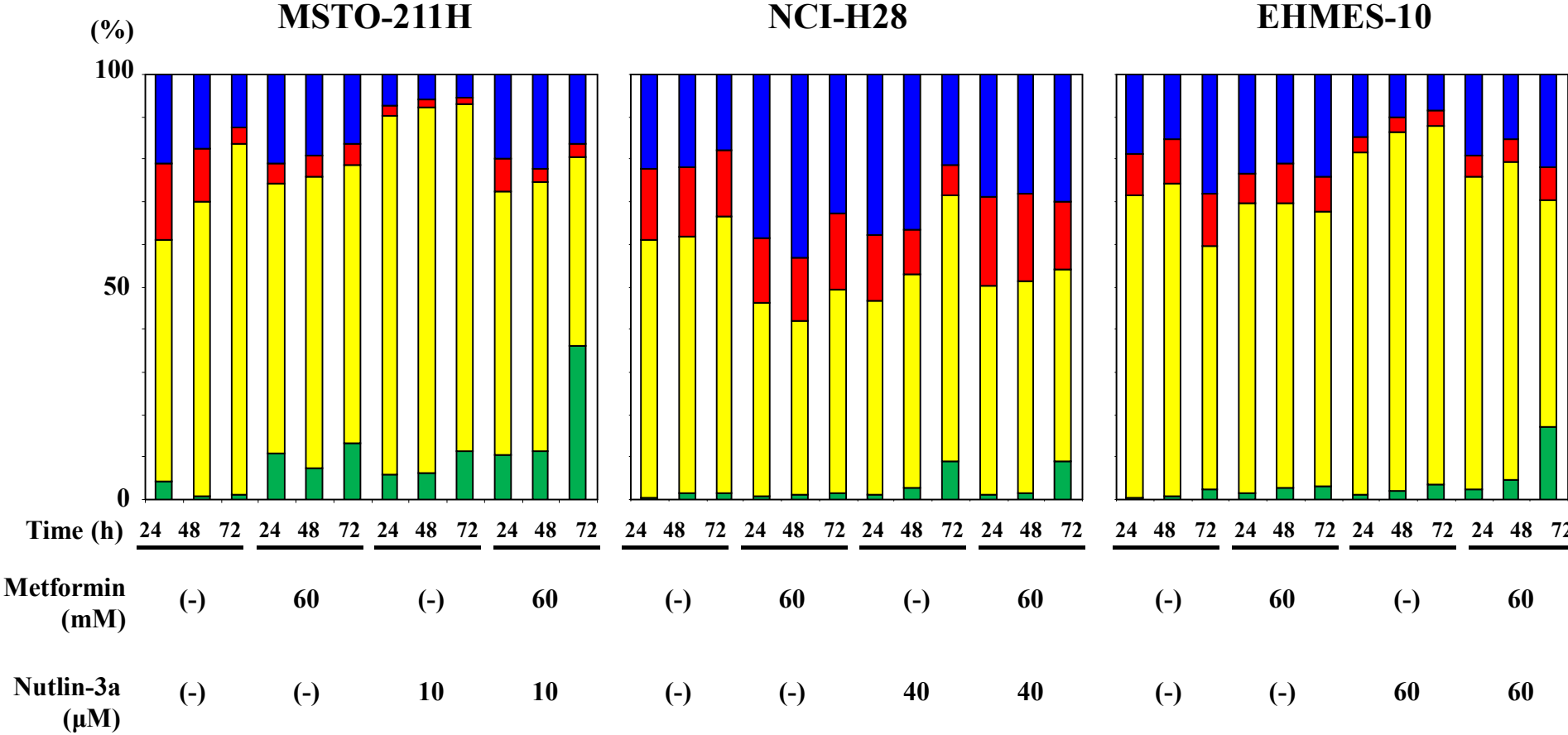

Supplement: Supplementary file 3 — Cell cycle changes caused by metformin and/or nutlin-3a. Cells were treated with metformin and nutlin-3a as indicated, and analyzed for the cell cycle with a flow cytometry. (PDF 138 kb) [file 12885_2017_3300_MOESM3_ESM.pdf]

## Supplementary Figure 3

(A)

MSTO-211H

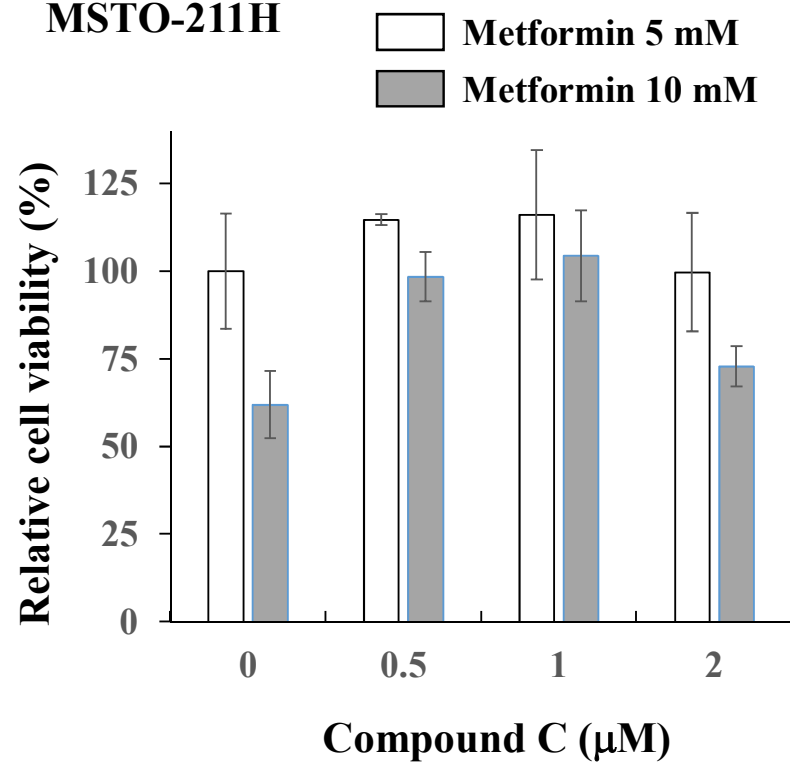

(B)

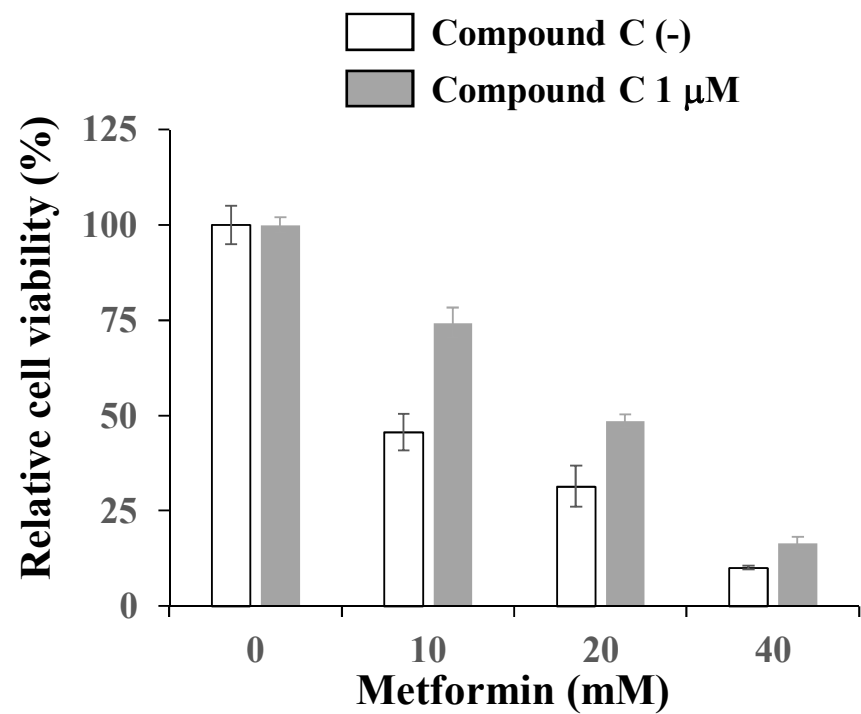

Supplement: Supplementary file 4 — AMPK inhibition blocked metformin-mediated suppression. MSTO-211H cells were treated with metformin and compound C as indicated for 4 days and relative cell viability was examined with the WST assay. Viability of cells treated with metformin at 5 mM but without compound C (A) and that without metformin or compound C (B) were shown as 100%. Averages and SEs are shown (n = 3). (PDF 72 kb) [file 12885_2017_3300_MOESM4_ESM.pdf]

## Supplementary Figure 4

(A)

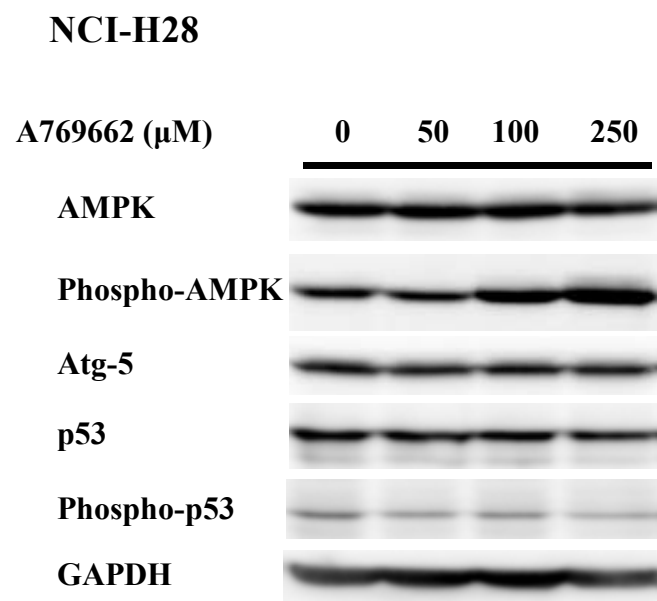

(B)

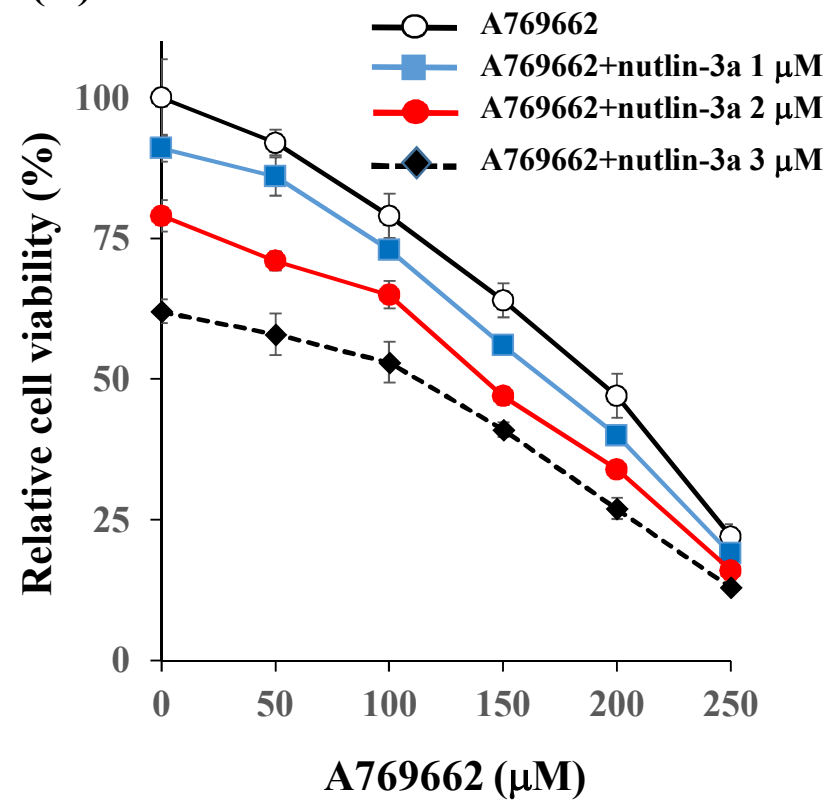

Supplement: Supplementary file 5 — (A) AMPK activation was irrelevant to Atg-5 and p53 expression. NCI-H28 cells treated with A769662, an AMPK activator, did not influence Atg-5, p53 or phosphorylated p53 levels. (B) An AMPK activator did not produce synergistic combinatory effects with nutlin-3a. NCI-H28 cells were treated with A769662 and nutlin-3a as indicated for 4 days and relative cell viability was examined with the WST assay. Averages and SEs are shown (n = 3). (PDF 106 kb) [file 12885_2017_3300_MOESM5_ESM.pdf]
