# Supplementary material for: Metformin produces growth inhibitory effects in combination with nutlin-3a on malignant mesothelioma through a cross-talk between mTOR and p53 pathways
Source: BMC Cancer. 2017 May 2;17:309. doi: 10.1186/s12885-017-3300-y (PMC5414226; doi:10.1186/s12885-017-3300-y)
Supplement: Supplementary file 6 — Combination Index by nutlin-3a and A769662. (DOCX 14 kb) [file 12885_2017_3300_MOESM6_ESM.docx]

Table S2. Combination Index by nutlin-3a and A769662

Nutlin-3a (μM) A769662 (μM) Fraction affected Combination Index

1 50 0.14 1.40

100 0.27 1.36

150 0.44 1.25

200 0.60 1.15

250 0.81 0.84

2 50 0.29 1.26

100 0.35 1.47

150 0.53 1.27

200 0.66 1.17

250 0.84 0.85

3 50 0.42 1.21

100 0.47 1.38

150 0.59 1.31

200 0.73 1.12

250 0.87 0.83

*NCI-H28 cells were treated with nultin-3a and A769662 and the combination indexes were calculated with CalcuSyn software. CI values are above 1 when Fa are between 0.2 and 0.8.
